# An Unusual Case of Membranoproliferative Glomerulonephritis: Is the Role of Vaccination in Immune Reactivation a Casual or Causal Effect?

**DOI:** 10.3390/reports8030141

**Published:** 2025-08-08

**Authors:** Celia Rodríguez Tudero, Alberto Martín Arribas, Marco Dominguez Davalos, Elena Jiménez Mayor, José Carlos De La Flor

**Affiliations:** 1 Surgery Doctoral Program, Faculty of Medicine, University of Salamanca, 37007 Salamanca, Spain; 2 Department of Nephrology, Hospital Universitario de Salamanca, 37007 Salamanca, Spain; amartinar@saludcastillayleon.es; 3 Department of Nephrology, Hospital Cayetano Heredia, Lima 15002, Peru; marco.dominguez.d@upch.pe; 4 Faculty of Medicine, Peruana Cayetano Heredia University, Lima 15002, Peru; 5 Departmente of Nephrology, Hospital San Pedro de Alcántara, 10001 Cáceres, Spain; elenajimenez215@gmail.com; 6 Department of Nephrology, Hospital Central Defense Gomez Ulla, 28047 Madrid, Spain; 7 Health Sciences Doctoral Program, Faculty of Medicine, Alcala University, 28805 Madrid, Spain; 8 Department of Medicine and Medical Specialties, Faculty of Medicine, Alcala University, 28805 Madrid, Spain

**Keywords:** MPGN (membranoproliferative glomerulonephritis), vaccination, kidney biopsy

## Abstract

**Background and Clinical Significance:** Membranoproliferative glomerulonephritis (MPGN) is a rare and heterogeneous pattern of immune-mediated glomerular injury, often associated with infections, autoimmune disorders, or monoclonal gammopathies. Idiopathic cases remain a diagnostic challenge and frequently require empirical immunosuppressive treatment. There is increasing interest in environmental triggers that may activate the immune system in genetically or immunologically predisposed individuals. We report an unusual case of idiopathic immune complex-mediated MPGN with a relapsing course potentially associated with vaccine-induced immune reactivation. **Case Presentation:** A 35-year-old male with no significant medical history aside from untreated dyslipidemia and active smoking presented with a hypertensive emergency and acute kidney injury (AKI). Laboratory investigations revealed nephrotic-range proteinuria, microscopic hematuria, and reduced estimated glomerular filtration rate (eGFR). Kidney biopsy demonstrated type I immune complex-mediated MPGN with a diffuse endocapillary proliferative pattern and granular subendothelial deposits (IgG+++, C3+++, C1q++). An extensive work-up ruled out secondary causes, supporting a diagnosis of idiopathic MPGN. Immunosuppressive therapy with corticosteroids and mycophenolate mofetil led to a partial clinical response. However, after receiving multiple vaccinations, the patient experienced clinical deterioration. A second biopsy revealed persistent proliferative changes and new deposits of IgM++, C4d++, and both kappa and lambda light chains. This prompted a reintroduction of immunosuppressive therapy, which resulted in subsequent clinical improvement. **Conclusions:** This case supports the hypothesis that vaccine-induced immune reactivation may serve as a potential trigger for disease relapse in idiopathic MPGN. Clinicians should remain alert to environmental stimuli that may influence disease activity in immune-mediated glomerulopathies. Further research is needed to elucidate the underlying immunopathogenic mechanisms.

## 1. Introduction and Clinical Significance

Membranoproliferative glomerulonephritis (MPGN) comprises a diverse spectrum of glomerular disorders marked by mesangial hypercellularity, thickening of the glomerular basement membrane, and the accumulation of immune complexes or complement components within mesangial and subendothelial regions. Morphologically, these alterations give rise to a lobulated glomerular configuration with a characteristic duplication of the basement membrane—commonly known as the “tram-track” pattern—observable via light and electron microscopy. Recent literature underscores the pivotal role of complement dysregulation and highlights ultrastructural features as key elements in refining the classification and understanding of MPGN and related C3 glomerulopathies [1].

With advances in the understanding of disease mechanisms, MPGN classification has shifted from a purely morphological system (types I, II, and III) to a pathophysiologic model. This modern approach distinguishes between immune complex–mediated MPGN and complement-mediated forms, collectively termed C3 glomerulopathy (C3G), which includes C3 glomerulonephritis (C3GN) and dense deposit disease (DDD) [2]. These subtypes differ in both clinical course and therapeutic implications.

C3 glomerulopathy (C3GN) frequently follows a progressive course leading to end-stage renal disease and is associated with a significant risk of recurrence following kidney transplantation. Recent reviews have shown that recurrence in the renal allograft remains a major clinical challenge, often resulting in graft dysfunction and eventual loss. In pediatric populations, C3GN recurrence post-transplant has been documented in up to two-thirds of cases, underscoring the need for close monitoring and individualized therapeutic strategies [3].

Furthermore, the coexistence of focal segmental glomerulosclerosis (FSGS) lesions within immune-mediated glomerulonephritis has gained increasing recognition. These lesions may represent secondary scarring from chronic glomerular injury or a concurrent pathogenic process, both of which carry adverse prognostic implications. FSGS lesions in the setting of immune glomerulopathies are associated with a poorer renal outcome and may influence therapeutic decision-making [4].

Definitive diagnosis requires a kidney biopsy with immunofluorescence and electron microscopy to clarify the underlying mechanism and guide clinical management.

The aim of this report is to describe a patient with immune complex–mediated MPGN of idiopathic etiology who experienced a clinical relapse temporally associated with the administration of multiple vaccinations. This case supports the hypothesis that vaccine-induced immune activation may act as a trigger in predisposed individuals.This case highlights the importance of considering environmental immune stimuli as potential triggers for disease activity in idiopathic glomerulopathies. The key finding is the temporal association between vaccination and MPGN relapse—a poorly documented phenomenon—which reinforces the need for individualized monitoring in patients with immune-mediated kidney diseases.

## 2. Case Presentation

A 35-year-old male carpenter with no known drug allergies presented to the emergency department after referral from his primary care center due to a hypertensive emergency (blood pressure 229/137 mmHg) and elevated serum creatinine identified on routine laboratory testing. His medical history included poorly controlled, untreated dyslipidemia; active smoking (15 pack-years); and habitual cannabis and alcohol use. He reported a 15-day history of persistent headache, unresponsive to paracetamol or nonsteroidal anti-inflammatory drugs (NSAIDs), with no accompanying chest pain, orthopnea, or paroxysmal nocturnal dyspnea. He occasionally experienced blurred vision in the right eye, and ophthalmologic evaluation revealed hypertensive retinopathy with periarcuate hemorrhages and cotton wool spots. He denied gross hematuria or tea-colored urine, and diuresis was preserved. On admission, vital signs revealed an oxygen saturation of 90% on room air and a heart rate of 90 bpm.

Initial laboratory investigations showed elevated serum creatinine 211.3 µmol/L with a reduced estimated glomerular filtration rate (eGFR) of 34 mL/min/1.73 m^2^. Urinalysis identified microscopic hematuria (198 erythrocytes/μL) with dysmorphic red blood cells in the urinary sediment and normal complement levels. He was diagnosed with non-oliguric AKI II in the context of nephritic syndrome, accompanied by nephrotic-range proteinuria and a hypertensive emergency [5]. A 24 h urine collection confirmed proteinuria of 7.19 g/day. Endocrine evaluations, including the hypothalamic–pituitary–adrenal axis, urinary catecholamines, and thyroid function, were within normal limits. An autoimmune work-up (ANA, anti-dsDNA, ANCA, anti-PLA2R, anti-GBM, anti-Ro, anti-La, and ASLO) was negative, as were complement studies. Immunoglobulin testing revealed low serum IgG levels. Serum and urine immunofixation, Bence Jones protein testing, and serum kappa/lambda free light chain ratios were unremarkable. The patient remained afebrile and showed no systemic symptoms.

Transthoracic echocardiography demonstrated preserved left ventricular ejection fraction (66%) with papillary muscle hypertrophy and elevated filling pressures. Renal ultrasound showed normal-sized kidneys without evidence of obstruction. Urinalysis was notable for marked proteinuria (>1000 mg/dL) and hematuria. The patient also presented with mild hypoalbuminemia (3 g/dL) in the absence of hepatic dysfunction or coagulopathy. Lipid profile revealed elevated total cholesterol and triglycerides, consistent with mixed dyslipidemia.

**Figure 1:**
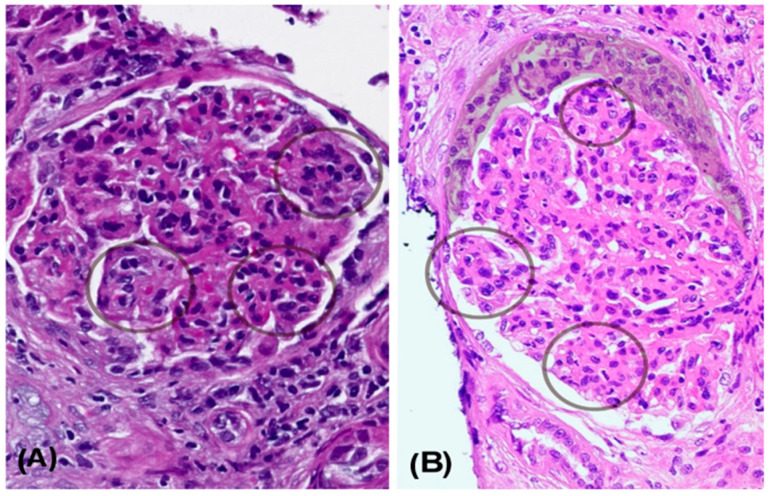
Progression of immune complex-mediated membranoproliferative glomerulonephritis. (**A**) H&E stain from the patient’s first kidney biopsy (November 2022), showing a lobulated glomerulus with diffuse involvement characterized by endothelial and inflammatory hypercellularity, collapse of glomerular capillary lumens, and a mesangiocapillary pattern (circles). (**B**) H&E stain from the second biopsy (November 2023), showing persistence of the mesangiocapillary pattern (circle), along with extracapillary proliferation and formation of a cellular crescent (shaded area).

**Figure 2:**
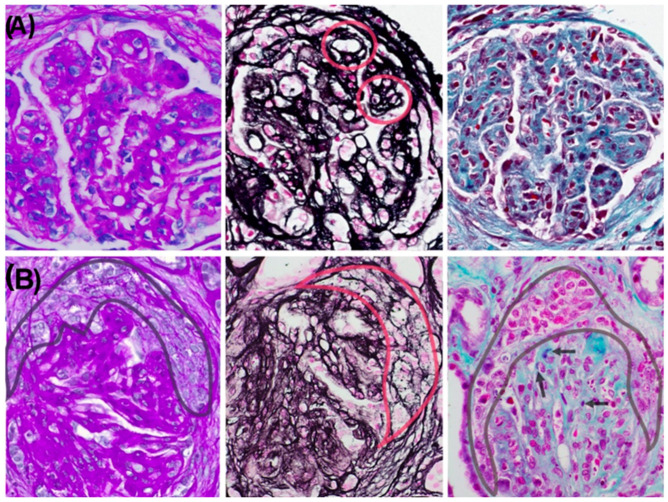
Composite of histochemical stains: PAS, silver stain, and Gomori trichrome (from left to right). (**A**) First biopsy (November 2022), where the stains highlight the lobulated glomerular pattern with PAS-positive mesangial expansion and basement membrane thickening with double contours (circles) on silver stain. (**B**) Second biopsy (November 2023) demonstrating preservation of the same injury pattern, with delineation of cellular crescents and fuchsinophilic deposits highlighted by Gomori trichrome stain (arrows).

**Figure 3:**
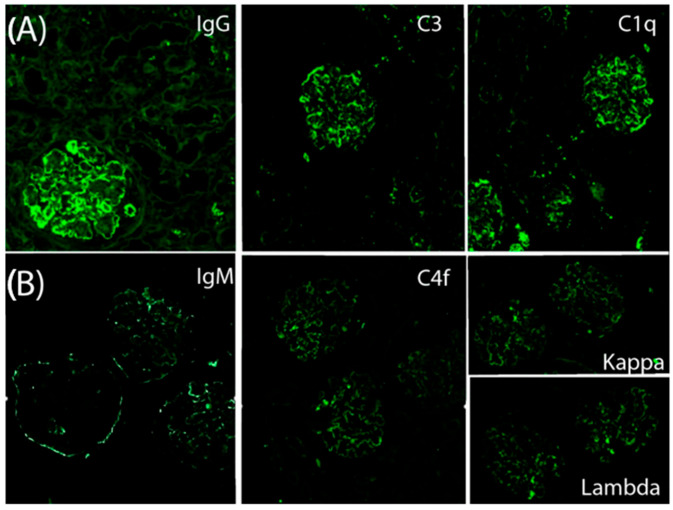
Composite of immunofluorescence (IF) stains. (**A**) First biopsy (November 2022) showing positivity for IgG++, C3+++, and C1q++. (**B**) Second biopsy (November 2023) showing persistent positivity for IgG+++, C3+++, and C1q++ (not shown), with additional positivity for IgM++, C4f++, kappa++, and lambda++ (shown). IF for IgA, fibrinogen, and albumin was negative in both biopsies.

A kidney biopsy performed in November 2022 included 31 glomeruli (Figure 1). Light microscopy revealed a diffuse and global endocapillary proliferative pattern with mesangiocapillary involvement and prominent subendothelial hypercellularity, consisting of 5–7 endothelial and mononuclear inflammatory cells per capillary loop. No crescents or fibrinoid necrosis were observed. The tubulointerstitial compartment showed scattered chronic lymphoplasmacytic infiltrates with eosinophils and minimal acute tubular injury. There was no significant interstitial fibrosis or evidence of viral cytopathic effect. The vascular structures displayed mild intimal hyperplasia without features of vasculitis. Optical microscopy showed lobulated glomeruli with 3% glomerulosclerosis (Figure 1A). Silver staining demonstrated basement membrane thickening with double contours (Figure 2A). Direct immunofluorescence revealed granular subendothelial and mesangial immune complex deposits (+++ IgG, +++ C3, ++ C1q; Figure 3A).

An extensive microbiologic, serologic, and immunologic work-up failed to identify a secondary cause, and the condition was classified as idiopathic membranoproliferative glomerulonephritis (MPGN). Chronic infections (hepatitis B and C), infective endocarditis, shunt nephritis, abscesses, autoimmune diseases (lupus, rheumatoid arthritis, Sjögren’s syndrome), monoclonal gammopathies, and lymphoplasmacytic disorders were systematically ruled out. In the absence of monoclonal immunoglobulin deposits or identifiable antigens, the disease was defined as immune complex–mediated idiopathic MPGN. Peak proteinuria reached 9.27 g/day. There was no evidence of light chain restriction, and staining for IgA, fibrinogen, and albumin was negative.

Initial treatment consisted of high-dose corticosteroid pulses (methylprednisolone 1 g × 3) and mycophenolate mofetil (MMF) at 1 g twice daily. This regimen led to progressive improvement in serum creatinine 159.1 µmol/L at discharge), although proteinuria (4.4 g/24 h) and microscopic hematuria persisted. In the subsequent months, proteinuria once again rose to nephrotic levels (15.86 g/24 h), prompting consideration of treatment intensification. The patient was evaluated for potential initiation of iptacopan therapy, which required an updated immunization schedule. Within 15 days, he received the following vaccines: meningococcal conjugate (A, C, W135, Y; Nimenrix), meningococcal B (Bexsero), influenza (Vaxigrip Tetra), 20-valent pneumococcal (Prevenar 20), COVID-19 (Pfizer), and Haemophilus influenzae type B (Hiberix). Shortly thereafter, the patient experienced a disease flare characterized by marked deterioration in renal function (creatinine 866.3 µmol/L), increased proteinuria (15 g/day), and significant weight gain.

He required two sessions of hemodialysis and received three additional pulses of methylprednisolone (1 g each). A second kidney biopsy revealed 20 glomeruli, 3 of which (6%) were globally sclerotic, while 12 (60%) exhibited either cellular (*n* = 9) or fibrocellular/fibrous crescents (*n* = 3). The glomeruli showed diffuse hypercellularity, mesangial expansion, endocapillary inflammation, and basement membrane double contouring. Special stains (PAS, silver, and Gomori) demonstrated nodular and segmental hyaline lesions consistent with active MPGN with extracapillary proliferation. Tubular findings included moderate atrophy (30%), focal acute tubular necrosis with signs of regeneration, and interstitial fibrosis involving 15% of the renal parenchyma. The interstitial infiltrate was patchy and mixed in composition (mononuclear cells with occasional neutrophils). Vascular changes included arteriolar hyalinosis and mild concentric intimal fibrosis with less than 20% luminal narrowing, without evidence of vasculitis. Direct immunofluorescence confirmed dense, granular subendothelial immune deposits positive for IgG (+++), IgM (++), C3 (+++), C1q (++), C4d (++), and both kappa and lambda light chains (++), while staining for IgA, fibrinogen, and albumin remained negative (Figure 3B).

At discharge, the patient continued MMF (1000 mg twice daily) and had already received the first intravenous pulse of cyclophosphamide, as part of a planned six-monthly pulse regimen (750 mg/month). Despite treatment, renal function declined (serum creatinine 785.9 µmol/L), and proteinuria remained massive (up to 19.88 g/24 h), with persistent microscopic hematuria. During outpatient follow-up (August 2024), he completed the remaining five monthly intravenous doses of cyclophosphamide, treatment was optimized to include MMF (1000 mg/day), low-dose prednisone (2.5 mg/day), and adjunctive therapies with nifedipine, losartan, dapagliflozin, spironolactone, bisoprolol, ezetimibe/atorvastatin, and prophylactic low molecular weight heparin (enoxaparin 20 mg/day). Nevertheless, the therapeutic response remained suboptimal, with creatinine levels ranging between 309.4 and 335.9 µmol/L and persistent nephrotic-range proteinuria. Serum IgG was markedly reduced (248 mg/dL), and rituximab was considered. However, its administration was controversial due to underlying hypogammaglobulinemia. Complement inhibition therapy was proposed under compassionate use, given an eGFR (CKD-EPI) of 24 mL/min/1.73 m^2^, but the patient was hesitant to receive the additional vaccinations required to initiate this treatment.

A temporal association was observed between the administration of multiple vaccines and the subsequent disease flare, raising the hypothesis that cumulative vaccine exposure may have triggered immune reactivation in a predisposed individual.

## 3. Discussion

We present the case of a relapse of immune complex–mediated membranoproliferative glomerulonephritis (IC-MPGN) following the administration of multiple vaccines, reviewing the clinical, histopathological, therapeutic, and disease course aspects.

MPGN represents a glomerular injury pattern seen in a heterogeneous group of disorders, predominantly affecting young adults., particularly within the spectrum of C3 glomerulopathy, is a well-recognized cause of progressive renal impairment that can ultimately lead to end-stage renal disease. Recent evidence highlights the high risk of chronic kidney function decline and graft failure, underscoring the importance of early diagnosis and tailored therapeutic approaches [2]. On light microscopy (LM), MPGN is defined by thickening or double-contour formation of the glomerular basement membrane (GBM) (“membrano”) accompanied by mesangial and/or endocapillary hypercellularity (“proliferative”) [6]. Based on IF findings, MPGN is classified into (IC-MPGN), complement-mediated MPGN (C3G), and MPGN negative for both immune complexes and complement deposits. IC-MPGN is an uncommon form of progressive kidney disease characterized by glomerular deposition of immune complexes and complement proteins [7].

Our patient initially presented with hypertension and impaired renal function. In a case series by Kovala et al., 98% of MPGN patients presented with hypertension and 65% with renal dysfunction. In addition, our patient had nephrotic-range proteinuria and microscopic hematuria. In the same series, 58% of patients had nephrotic-range proteinuria, and 82% presented with hematuria [8].

Renal biopsy revealed a lobular glomerular architecture with mesangial and endocapillary hypercellularity, consistent with IC-MPGN. IF demonstrated strong staining for IgG, C3, and C1q, supporting this diagnosis. Given the rarity of idiopathic IC-MPGN, a comprehensive diagnostic evaluation was undertaken to exclude secondary causes such as chronic infections, autoimmune diseases, monoclonal gammopathies, and neoplastic conditions [9]. In the absence of any identifiable etiology, the condition was classified as idiopathic. However, some experts have proposed that truly idiopathic IC-MPGN is becoming increasingly rare, as many of these cases may harbor underlying pathogenic mechanisms that remain subclinical or undetectable at the time of diagnosis [7].

According to the 2021 KDIGO guidelines on glomerular diseases, patients with idiopathic IC-MPGN, reduced renal function (in the absence of crescents), active urinary sediment, and nephrotic-range proteinuria should receive treatment with glucocorticoids and immunosuppressive therapy alongside supportive measures [10]. In our case, corticosteroids were administered initially with limited response, which led to the introduction of MMF at week 12. This resulted in improved renal function and partial reduction in proteinuria, although proteinuria levels remained within the nephrotic range.

The patient updated his vaccination schedule in preparation for inclusion in a clinical trial involving iptacopan, an alternative pathway complement inhibitor. He received vaccinations against Haemophilus influenzae, influenza, pneumococcus, COVID-19, and meningococcus. One month after vaccination, he developed anasarca, abrupt deterioration in renal function, and increased proteinuria, which prompted a repeat kidney biopsy.

In contrast to the initial biopsy, the second LM study revealed extracapillary proliferation (crescents) in 60% of glomeruli. While the first biopsy had shown typical features of type I MPGN, the second episode presented with a much more aggressive histological pattern consistent with crescentic transformation. This progression underscores a rare but documented phenomenon in MPGN, where a stable or slowly progressive disease may evolve into rapidly progressive glomerulonephritis (RPGN) with extensive crescent formation. Similar cases have been described in the literature, including a young adult patient initially diagnosed with idiopathic MPGN who later developed crescentic GN confirmed by sequential biopsies [11] and a pediatric case with type I MPGN presenting with diffuse crescents at relapse that responded to intensive immunosuppressive therapy [12]. These findings highlight the potential for histopathological transformation in MPGN and the importance of re-biopsy in the context of clinical deterioration.

The second IF study demonstrated strong positivity for IgM, IgG, C3, C1q, C4d, as well as both kappa and lambda light chains, confirming persistent IC-MPGN with an increased burden of immune deposits. A comprehensive reevaluation for secondary etiologies remained negative. Notably, the patient had recently received multiple vaccinations, which may have acted as a potential trigger for the disease relapse.

The literature regarding vaccine-associated glomerular disease remains conflicting. Although rare, several case reports have described associations between vaccinations and renal complications, including glomerulopathies and acute kidney injury [13]. In our case, the patient had received the Pfizer-BioNTech COVID-19 mRNA vaccine. Göndör et al. reported a similar case in which stable chronic MPGN progressed to crescentic glomerulonephritis following administration of a COVID-19 mRNA vaccine [14]. In 2025, Pethő et al. published a case series of six patients who developed de novo glomerulonephritis after administration of the COVID-19 mRNA vaccine. Three of the six developed a minimal change pattern, but none developed MPGN. During a follow-up of more than two years, remission occurred in five patients, and glomerulonephritis persisted in one of them [15]. mRNA vaccines are known to induce robust cellular and humoral immune responses. One proposed mechanism linking vaccination to glomerular disease involves immune activation of both the innate and adaptive systems [16]

Although no cases of MPGN have been reported following influenza vaccination, multiple instances of systemic vasculitis have been described [17], including leukocytoclastic vasculitis with pauci-immune crescentic glomerulonephritis and necrotizing glomerulonephritis [18]. Kikuchi et al. reported a case of minimal change disease and acute interstitial nephritis in a 67-year-old woman following pneumococcal vaccination [19]. A 2003 study suggested an increased risk of nephrotic syndrome relapse in children after receiving the meningococcal C conjugate vaccine (MCCV) [20]; however, subsequent studies have failed to confirm this association [21]. Reports of glomerular disease following Haemophilus influenzae type B vaccination are virtually nonexistent.

Although a definitive causal relationship cannot be established, the temporal association between vaccination and disease flare in this patient suggests a potential immune reactivation triggered by cumulative vaccine exposure. Given the number of vaccines administered, it is difficult to identify a single causative agent or to rule out a synergistic effect.

Crescentic transformation of MPGN is frequently associated with rapidly progressive glomerulonephritis (RPGN) [22], defined as a ≥50% decline in eGFR within three months [10]. RPGN may occur at initial presentation or during disease progression [23]. According to KDIGO 2021 guidelines, idiopathic crescentic IC-MPGN should be treated with high-dose glucocorticoids and cyclophosphamide, following therapeutic regimens similar to those for ANCA-associated vasculitis [5,10].

Our patient received corticosteroids, cyclophosphamide, and MMF, with a poor clinical response. Rituximab was considered but contraindicated due to persistent IgG hypogammaglobulinemia. The patient underwent close follow-up in the outpatient setting. At six months, there was no recovery of renal function. Proteinuria persisted between 10 and 15 g/24 h despite immunosuppressive and supportive treatment. Serum creatinine reached approximately 335 µmol/L. Given the poor clinical course, renal replacement therapy was indicated, and the patient opted for peritoneal dialysis.

A clinical trial (NCT05755386) is currently underway to evaluate the efficacy of iptacopan—a potent oral factor B inhibitor targeting the alternative complement pathway—in patients with idiopathic IC-MPGN. Although the study excludes crescentic forms, it may pave the way for future therapeutic options in more aggressive IC-MPGN phenotypes [24].

Renal prognosis in crescentic MPGN has historically been poor. While a minority of patients respond to intensive immunosuppression or plasmapheresis, many progress to end-stage kidney disease and exhibit high rates of disease recurrence and graft rejection following transplantation. Our patient presented with several unfavorable prognostic indicators, including impaired renal function at baseline, nephritic syndrome, crescent formation, and tubular atrophy involving 30% of glomeruli.

While this case report has some limitations, including the absence of long-term follow-up and mechanistic studies, it provides valuable clinical and histopathological documentation of a rare crescentic transformation of IC-MPGN temporally associated with multiple vaccinations. This report may offer useful insights for clinicians and contribute to future research on immune-mediated glomerular diseases.

## 4. Conclusions

We report the clinical, laboratory, and histopathological progression of a patient with previously stable IC-MPGN who experienced a severe crescentic relapse following the administration of multiple vaccines within a short period. While a definitive causal relationship with any individual vaccine cannot be established, the temporal proximity of several vaccinations may have acted as a cumulative immune stimulus in a predisposed host, potentially aggravating the underlying dysregulated complement pathway. We explicitly clarify that this is a hypothesis and should not be interpreted as attributing causality to any specific vaccine. Among the vaccines administered, COVID-19 and influenza vaccines have been more frequently associated with glomerular diseases in the published literature; however, this observation remains speculative. Further research is warranted to elucidate the potential mechanisms linking immune activation, including vaccination, to disease flares in patients with MPGN.

## Data Availability

No new data were created or analyzed in this study. The data used to support the findings of this study are available from the corresponding author on request (Contact C.R.T, crodrigueztudero@usal.es).
